# Optimization of Immunofluorescent Detection of Bone Marrow Disseminated Tumor Cells

**DOI:** 10.1186/s12575-018-0078-5

**Published:** 2018-07-01

**Authors:** Haley D. Axelrod, Kenneth J. Pienta, Kenneth C. Valkenburg

**Affiliations:** 1 The James Buchanan Brady Urological Institute, Johns Hopkins University School of Medicine, 600 North Wolfe St., Marburg Building Room 121, Baltimore, MD 21287 USA; 2 The Cellular and Molecular Medicine Program, Johns Hopkins School of Medicine, 600 North Wolfe St., Marburg Building Room 121, Baltimore, MD 21287 USA

**Keywords:** Immunofluorescence, Cancer, CTC, DTC, Bone Marrow, Blood, Detection

## Abstract

**Background:**

Cancer metastasis is the primary cause of cancer-related deaths and remains incurable. Current clinical methods for predicting metastatic recurrence are not sensitive enough to detect individual cancer cells in the body; therefore, current efforts are directed toward liquid biopsy-based assays to capture circulating and disseminated tumor cells (CTCs and DTCs) in the blood and bone marrow, respectively. The most promising strategy is fluorescence-based immunostaining using cancer cell-specific markers. However, despite recent efforts to develop robust processing and staining platforms, results from these platforms have been discordant among groups, particularly for DTC detection. While the choice of cancer cell-specific markers is a large factor in this discordance, we have found that marker-independent factors causing false signal are just as critical to consider. Bone marrow is particularly challenging to analyze by immunostaining because endogenous immune cell properties and bone marrow matrix components typically generate false staining. For immunostaining of whole tumor tissue containing ample cancer cells, this background staining can be overcome. Application of fluorescent-based staining for rare cells, however, is easily jeopardized by immune cells and autofluorescence that lead to false signal.

**Results:**

We have specifically found two types of background staining in bone marrow samples: autofluorescence of the tissue and non-specific binding of secondary antibodies. We systematically optimized a basic immunofluorescence protocol to eliminate this background using cancer cells spiked into human bone marrow. This enhanced the specificity of automated scanning detection software. Our optimized protocol also outperformed a commercial rare cell detection protocol in detecting candidate DTCs from metastatic patient bone marrow.

**Conclusions:**

Robust optimization to increase the signal-to-noise ratio of immunofluorescent staining of bone marrow is required in order to achieve the necessary sensitivity and specificity for rare cell detection. Background immunofluorescent staining in bone marrow causes uncertainty and inconsistency among investigators, which can be overcome by systematically addressing each contributing source. Our optimized assay eliminates sources of background signal, and is adaptable to automated staining platforms for high throughput analysis.

**Electronic supplementary material:**

The online version of this article (10.1186/s12575-018-0078-5) contains supplementary material, which is available to authorized users.

## Background

Approximately 600,000 cancer-related deaths occur in the U.S. every year, and nearly all are due to metastasis [1–3]. Once metastatic, cancer is usually incurable; this has led current efforts to focus on early detection of cancer cells by liquid biopsy. However, the accurate detection and characterization of circulating tumor cells (CTCs) in the blood and disseminated tumor cells (DTCs) in the bone marrow (BM) has proven to be challenging. Despite widespread efforts to design assays with the necessary sensitivity and specificity to capture extremely rare cells (1 CTC per ten million white blood cells) there remains only one FDA-approved image-based immunofluorescence (IF) platform, limited to CTC detection [4–6]. In addition to IF-based rare cell detection, another widely used strategy is based on real time PCR (RT-PCR). RT-PCR methods do not capture individual cancer cell heterogeneity and rely on RNA expression, while IF assays are not as sensitive. Each strategy bears its own limitations, but IF provides several advantages over RT-PCR detection. IF of blood and BM smears allows for the characterization of individual cells at the protein level, where expression does not always correlate with RNA expression [7]. In addition to being able to analyze the expression of multiple proteins, information on protein localization and cell size, shape, and aggregate behavior can be assessed. Combining these features would provide a more informative landscape of the disease to aid in diagnosis, prognosis, and treatment strategies, and thus IF staining has emerged as the most appealing rare cell detection strategy. However, current IF procedures for staining CTCs and DTCs vary widely and have not yielded consistent results [4, 5].

While pathologists successfully use chromogenic immunostaining to detect the presence of cancer in many types of tissue with few limitations, when it comes to detecting rare cells these limitations become unacceptable. Compared to chromogenic staining, IF introduces additional factors that need to be controlled for such as microscope exposure time, brightness and contrast settings, photobleaching, and autofluorescence. Improper control of these factors can lead to false negative or positive signal. These misleading effects can be easily avoided in samples where the target cell population is abundant, but can pose complications when trying to detect rare cells. These problems become further amplified when staining and imaging BM, specifically. BM contains a large number of immune cells which, by nature, bind antibodies and engulf foreign particulates. This is largely facilitated by fragment crystallizable (Fc) receptors on the surface of many immune cells. Therefore, when immune cells are present in the specimen being stained it is crucial that appropriate blocking factors be included that inhibits Fc receptor binding, prior to the addition of any antibody. Immune cells also tend to be more autofluorescent than other cell types. This has been observed in large macrophages when exposed to 488 nm light, due to their high content of flavoprotein-associated granules [8, 9]. Another source of blood and BM autofluorescence is lipofuscin, a product of oxidized proteins and lipids commonly found in macrophages and red blood cells and which fluoresces in most channels [10–12]. Importantly, the BM contains additional extracellular matrix components such as collagen and other autofluorescent non-collagenous proteins which make IF staining of BM more challenging than blood [13–16]. Current IF protocols for the detection of CTCs and DTCs do not consider all of the aforementioned sources of false signal, and this is likely a major factor in the inconsistency of reports that utilize different procedures. It is crucial to consider these potential sources when trying to identify DTCs. Since bone marrow DTCs have not yet been well characterized, it is difficult to judge true positive signal based on histopathological or protein expression traits. Some rare cell detection strategies rely on filtering out immune cells by size, charge, and/or marker exclusion before being processed onto a slide for staining, but these methods run the risk of occasionally filtering out cancer cells [4, 7, 17]. For techniques that do not involve a physical selection step, thorough optimization has been limited by the constraints of autostaining platforms, which are necessary when processing large volumes to find rare events.

We had initially set out to test various cancer cell-specific markers for the detection of DTCs using several different protocols, but found a remarkable number of epithelial marker-positive cells in cancer-negative control samples, in addition to inconsistencies in overall staining. In order to eliminate these uncertainties and develop a reliable staining protocol, we assessed each step of a basic IF protocol for sources contributing to false signal in BM samples. We used a pan-cytokeratin antibody to detect prostate cancer cells spiked into human BM, as this epithelial marker has been consistently used for rare cancer cell detection across a variety of platforms [18–21]. While we recognize that determining cancer cell-specific markers will be crucial in the accurate detection of rare cells, it is first necessary that the staining procedure results in minimal background and consistency across samples, so that cancer-specific markers can be accurately assessed for their specificity and best signal-to-noise ratio. Initially, we observed two main sources of background in particular: tissue autofluorescence and non-specific binding of secondary antibodies. We then systematically optimized the basic IF protocol to increase signal-to-noise ratio of true staining so as to eliminate background signal and to produce an image in which cancer cells were easily identifiable using automated detection software. The ultimate goal of this study was to adapt our optimized protocol for use in automated staining platforms to reliably detect DTCs in clinical samples. While our protocol was optimized using BM samples, we anticipate the staining of blood samples to be of equal or greater quality due to the reduced background staining from the immune cell component.

## Methods

For a comprehensive list of reagent vendors and catalog numbers, see Additional file 1: Table S1.

### Cell Culture and Cancer Cell Spiking

LNCaP prostate cancer cells (ATCC) were maintained in RPMI supplemented with 10% FBS and 1% penicillin/streptomycin. Before spiking into bone marrow, LNCaP cells were seeded with 5 nM R1881 (Sigma, #R0908) in media with charcoal-stripped FBS for two days. Cells were harvested using non-enzymatic cell dissociation buffer (ThermoFisher, #13151014), resuspended in media, washed in PBS, and then counted using the Countess II FL Automated Cell Counter (ThermoFisher). LNCaP cells were spiked into bone marrow at 1000 LNCaP cells per 1.5 million white blood cells (WBCs). Bone marrow was collected from patients following signed written formal consent approved by the Johns Hopkins Office of Human Subjects Research Institutional Review Board.

### Bone Marrow Processing for Adhesion Slides

Human BM aspirate was collected into CellSave Preservative Tubes (Veridex/Janssen Diagnostics, #7900005) from the pubic bone of low grade prostate cancer patients at the time of radical prostatectomy (see Additional file 1: Table S2 for specific clinical information). The BM was processed within 48 h of collection. 5 mL of aspirate was added to 45 mL of ACK Lysing Buffer (Quality Biological, #118–156-101) and incubated on a rotator for 10 min and then spun down at 1500×g for 10 min. Pelleted cells were resuspended in 5 mL of PBS and counted using a WBC counter (HemoCue®). A desired volume corresponding to 1.5 million WBCs per square on a Marienfeld Adhesion Slide (Azer Scientific, Inc., #ES0909101) was spun down again and resuspended in PBS corresponding to 300 μL per square. 1000 LNCaP cells were added to spiked samples before spinning down. Resuspended cells were pipetted onto slides and incubated at 37 °C for 1 h to promote adhesion to slides. Excess PBS was decanted, and slides were then incubated with 4% paraformaldehyde (ThermoFisher, #28908) for either 10 or 30 min. After fixation, slides were washed in PBS before. To dehydrate, slides were incubated in 50% ethanol for 5 min, 70% ethanol for 5 min, and then 100% for 5 min. Slides were transferred to storage tubes (Fisher Scientific, #22–038-399) containing 100% ethanol and were placed at − 20 °C for long-term storage. All steps were performed at room temperature unless otherwise indicated.

### Bone Marrow Processing for Plus Slides Using the RareCyte System

Six mL of BM from metastatic prostate cancer patients (see Additional file 1: Table S3 for specific clinical information) was harvested in RareCyte® BM tubes. Each tube was inverted 8 times. Each BM sample was filtered through a 100 μM Nylon cell strainer (Falcon, #352360). WBC counts were performed using the HemoCue® WBC counter. 7.5 mL of PB or 4 mL of BM was pipetted into an AccuCyte® Separation Tube, which was then inserted into a centrifuge adapter. Each sample was centrifuged at room temperature for 25 min at 5,200×g. Using the CyteSealer®, a ring was applied around each Separation Tube. Plasma was saved and stored in − 80 °C. 4 mL of AccuCyte® Displacement Fluid was placed into the Separation Tube, which was then removed by the insertion of the EpiCollector® into the Separation Tube. A locking clip was attached to the Separation Tube. The AccuCyte® Shield Tube was removed and replaced with the AccuCyte® Isolation Tube. 160 μL of AccuCyte® Isolation Fluid was pipetted into the Isolation Tube and each sample was centrifuged at room temperature for 20 min at 1000×g. The Isolation Tube was removed and 760 μL of AccuCyte® Transfer Fluid was added to resuspend the pellet in the Isolation Tube. 95 μL of the resuspended pellet was spread across 24 Superfrost® Plus slides and air-dried. Slides were then stored at − 20^°^C.

### Thawing Plus Slides for Optimized Protocol Staining

Frozen plus slides were thawed for 10 min at room temperature, and then fixed with 4% paraformaldehyde for 10 min. They were then washed in PBS for 3 min, and then washed in PBST two times for 3 min each. Staining was then initiated starting with the 0.5% Triton X-100 permeabilization step in the optimized protocol.

### Basic Staining Protocol

All steps were performed at room temperature unless otherwise indicated. All reagents were applied by gently pipetting 300 μL onto each square of the Adhesion slide and placing in a humidity chamber. First, stored slides were rehydrated by incubating in 70% ethanol for 5 min, 50% ethanol for 5 min, then PBS for 10 min. Next, cells were permeabilized with 0.5% Triton X-100 diluted in PBS for 20 min. Slides were then washed by dipping into fresh PBS three times and blocked by adding 10% goat serum for 20 min at 37 °C. Primary antibodies were diluted in 10% goat serum and applied to slides for 30 min at 37 °C after decanting block. Slides were washed in PBS three times, incubated in secondary antibodies diluted 1:1000 in PBS for 30 min at 37 °C in the dark, and washed another three times. Finally, glass coverslips were mounted onto the slides using DAPI-containing mounting media, and were left to cure overnight in the dark.

### Optimized Staining Protocol

All steps were performed at room temperature unless otherwise indicated. All reagents were applied by gently pipetting 300 μL onto each square of the Adhesion slide and placing in a humidity chamber. Stored slides were rehydrated by incubating in 70% ethanol for 5 min, 50% ethanol for 5 min, then PBS for 5 min. Thorough washing was performed by placing slides in fresh PBST two times for 3 min each.

Cells were permeabilized by incubating in 0.5% Triton X-100 diluted in PBS for 20 min. Slides were washed two times in PBST as indicated above, and one time in PBS. For the first blocking step slides were exposed to TrueBlack™ diluted fresh in 70% ethanol for 1 min, then washed in PBS two times and in PBST one time. Additional blocking steps were performed by incubating slides in Image-iT™ FX Signal Enhancer for 30 min, then decanting and adding 5% BSA diluted in PBS spiked with human Fc receptor blocker at 5 μL per 1 million cells for 30 min. BSA/FcR blocker was then decanted and primary antibody diluted in 5% BSA was applied to slides for 1 h. Slides were washed in PBST three times, then incubated in secondary antibodies diluted in PBS for 45 min in the dark. Whole secondary antibodies were diluted at 1:2500 and F(ab) fragment secondary antibodies were diluted at 1:800. After washing slides in PSBT three times, DAPI diluted in PBS at 1 mg/L was added for 5 min in the dark. Slides were washed in PBST three times before mounting the coverslip using mounting media without DAPI, and were left to cure overnight in the dark.

### RareCyte Manual Staining Protocol

Frozen bone marrow smears on plus slides were thawed on the benchtop at room temperature for 5–10 min, and then fixed in 10% NBF for 1 h. Slides were washed in TBS 2 times for 5 min, then placed in PBS before performing heat-mediated antigen retrieval in Tris-HCl buffer, pH 10, for 6 min at 75 °C. Slides were transferred to fresh PBS for 5 min to cool, and were then washed four times in Dako buffer (Dako, #K8007) by directly pipetting buffer onto each slide and decanting. Reagent 1 was then added for 30 min, followed by four washes in Dako buffer. Slides were then incubated in Reagent 2A for 40 min, followed by another set of washes, incubated in Reagent 2B for 40 min, and then washed again. Reagent 3 was spiked fresh with anti-CD11b-PE, CD14-PE, and CD34-PE at 1:200 and incubated on slides for 30 min, followed by a final set of washes. Slides were then transferred to PBS and cover-slipped with RareCyte mountant to sit overnight at room temperature in the dark.

### Heat-Mediated Antigen Retrieval

Instead of incubating slides in Triton X-100, slides were placed into microwave-safe containers (PerkinElmer, #STJAR4) with either citrate-based Antigen Unmasking Solution (Vector Laboratories, #H-3300), Target Retrieval solution (Dako, #S1699), or EDTA solution (ThermoFisher, #005500) diluted to 1X in distilled water. Slides were microwaved at 100% power for 50 s, and then 20% power for 15 min. Slides were then placed on the benchtop to cool at room temperature for 15 min.

### Manual Imaging of Slides

Slides were loaded onto a Carl Zeiss AxioImager.Z2 equipped with a PhotoFluor LM-75 halide light source (89 North), a CoolCube 2 m monochrome camera (MetaSystems, #H-0310-013-MS), and a motorized 8 slide stage using a manual movement control system (MMC) (MetaSystems, V2.4.5). Images were captured with the Isis Fluorescence Imaging Platform (MetaSystems, V5.8.5) using a Zeiss EC Plan-Neofluar 40×/0.75 M27 objective and a Zeiss Plan-Achromat 20×/0.8 M27 objective. Slides from the same experiment were imaged using the same settings (exposure time, upper threshold, lower threshold). Nuclear staining with 4′6-Diamidino-2-Phenylindole (DAPI) was visualized using excitation 359 and emission 461 (custom DAPI filter set); AF488 was visualized with excitation 495/25 and emission 537/29 (Chroma, #49303); AF555 was visualized with excitation 550/25 and emission 605/70 (Zeiss, Filter Set 43 HE); AF647 was visualized with excitation 640/30 and emission 690/50 (Chroma, #49009).

### Imaging Slides Using Metafer Scanning Software

The number of false and true positive cancer cells in bone marrow slides were counted using the Metafer5 (MetaSystems, V3.11.8) automated scanning system. Slides were loaded onto the motorized 8 slide stage of the microscope indicated above. Scans were performed using a Zeiss Plan Apochromat 10×/0.45 objective to scan in the XY plane, and one Z focus plane was selected automatically for every tile using a course focus of 10 planes of 7.5 μm and then a fine focus of 2 μm distance. Slides from the same experiment were scanned using the same area and exposure time for each channel with a 5.0 camera gain. Minimum/maximum exposure times (seconds) for each channel in Fig. 5 were: DAPI 0.0037/0.0111, AF488 0.0192/0.12, AF555 0.0192/0.04. Exposure times for scans in Fig. 7 were: DAPI 0.0092/0.0159, AF488: 0.0092/0.24, AF555: 0.0092/0.08.

### Metastatic Patient Sample Collection, Processing, and Analysis

Three patient bone marrow aspirates were collected adjacent to the site of a metastatic prostate cancer lesion (see Additional file 1: Table S3) and were processed onto plus slides using the RareCyte system. Slides were stained using the RareCyte manual staining protocol or the optimized protocol. The same defined region was scanned for each slide. Manual selection of the populated gallery after automated scanning detection was performed as in Fig. 5b. The scan could not be completed for Patient 2 BM stained with the RareCyte protocol due to the event limit being reached. Number of total populated cells was thus estimated based on proportion of area that was scanned (see Table 1).

Slides from the same experiment were also subjected to the same criteria for calling candidate CK+ cells. Criteria for manual selection after automated selection is described in the results section. Final cell galleries were exported using Adobe Acrobat.

## Results

### Sources of Background Signal

**Fig. 1:**
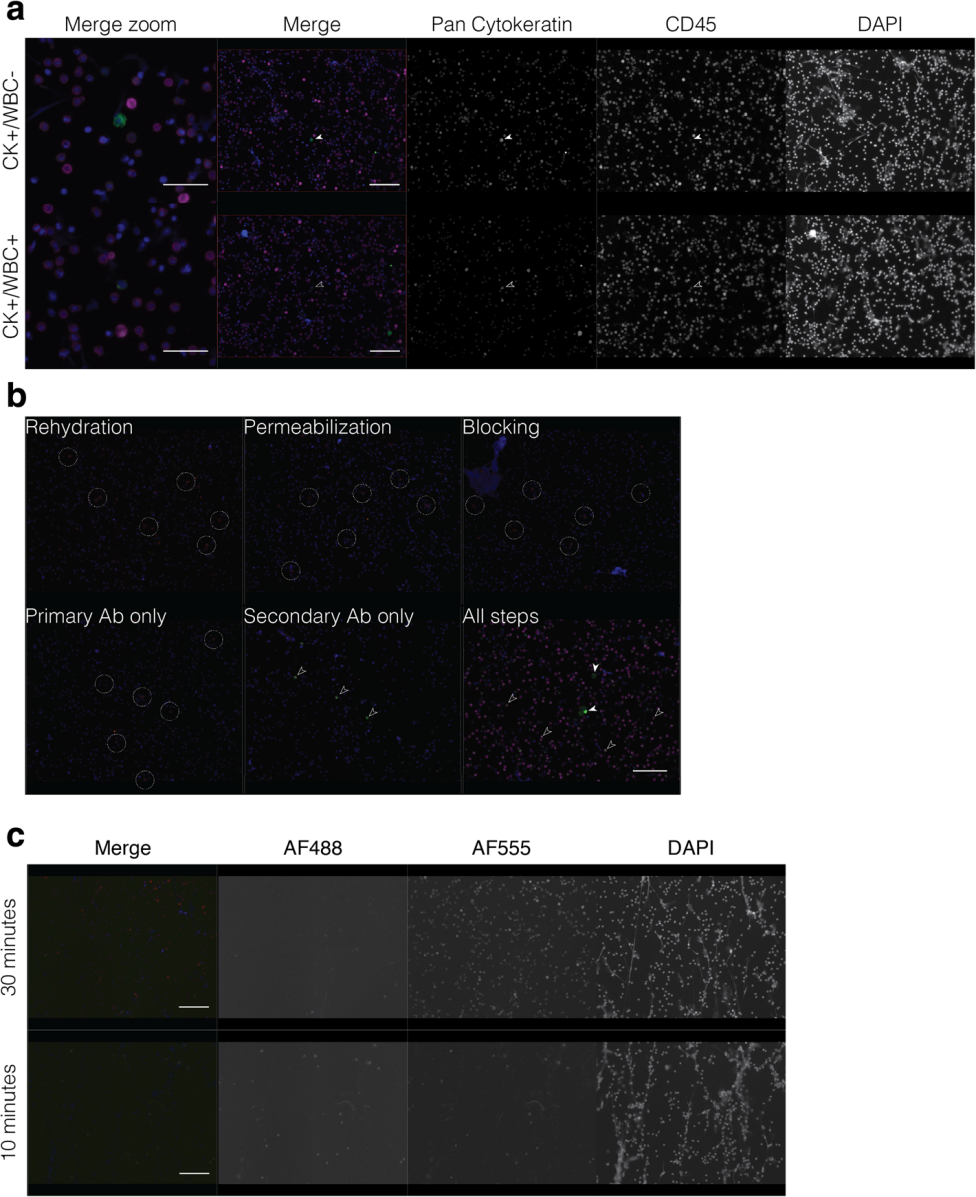
Identification of sources contributing to background staining in cancer cell-spiked human bone marrow. **a** Demonstration of true pan cytokeratin+ and white blood cell- (CK + WBC-) putative cancer cell and CK + WBC+ putative WBC using the basic protocol. **b** Autofluorescence and non-specific secondary antibody binding are evident after rehydration of the slides and secondary antibody incubation, respectively. After all steps are performed, CK staining is detected by AlexaFluor 488 (AF488, green) and CD45 by AF647 (magenta). All images capture signal from all channels, although there is no staining in the AF555 channel. **c** Autofluorescence in primary unstained bone marrow fixed with 4% PFA for 30 min or 10 min. Filled white arrowheads point to true positive staining. Dashed circles indicate background staining on representative cells due to autofluorescence, while open solid arrowheads indicate false staining due to non-specific binding of secondary antibody. Scale bar = 20 μm for merge zoom image, 50 μm for merged and respective individual channel images in the remainder of the figure

LNCaP prostate cancer cells spiked into human BM were stained with pan cytokeratin (CK) and CD45 (white blood cell (WBC) marker), resulting in the detection of expected CK + WBC- cells but also CK + WBC+ cells (Fig. 1a). To determine the cause(s) of double positive staining, we cover-slipped and imaged slides after each step in our basic IF protocol [7]. This included imaging after fixation, blocking, primary antibody, secondary antibody only (no primary), and secondary antibody (after primary) steps. We observed autofluorescence immediately after fixation, where a fraction of cells was autofluorescent in every channel, indicating autofluorescence of the BM itself (Fig. 1b). Incubation with unlabeled primary antibody did not further affect signal, but incubation with fluorescently labeled secondary antibody (hereafter referred to simply as secondary antibody) led to substantial signal in a fraction of cells, independent of the BM autofluorescence. In BM samples spiked with cancer cells and stained with both primary and secondary antibodies, CK + WBC- cells were evident as in Fig. 1a. However, the CK staining intensity on these cells was not uniform across all spiked cancer cells, and in some cases, was indistinguishable from the CK + WBC+ signal. The observed autofluorescence in the initial step after fixation was not surprising, as aldehyde-based fixatives are known to produce autofluorescence. Therefore, we decreased the fixation time to 10 min from 30 min. This was able to reduce autofluorescence without compromising the cells and their subsequent staining (Fig. 1c).

### Antigen Retrieval Optimization

**Fig. 2:**
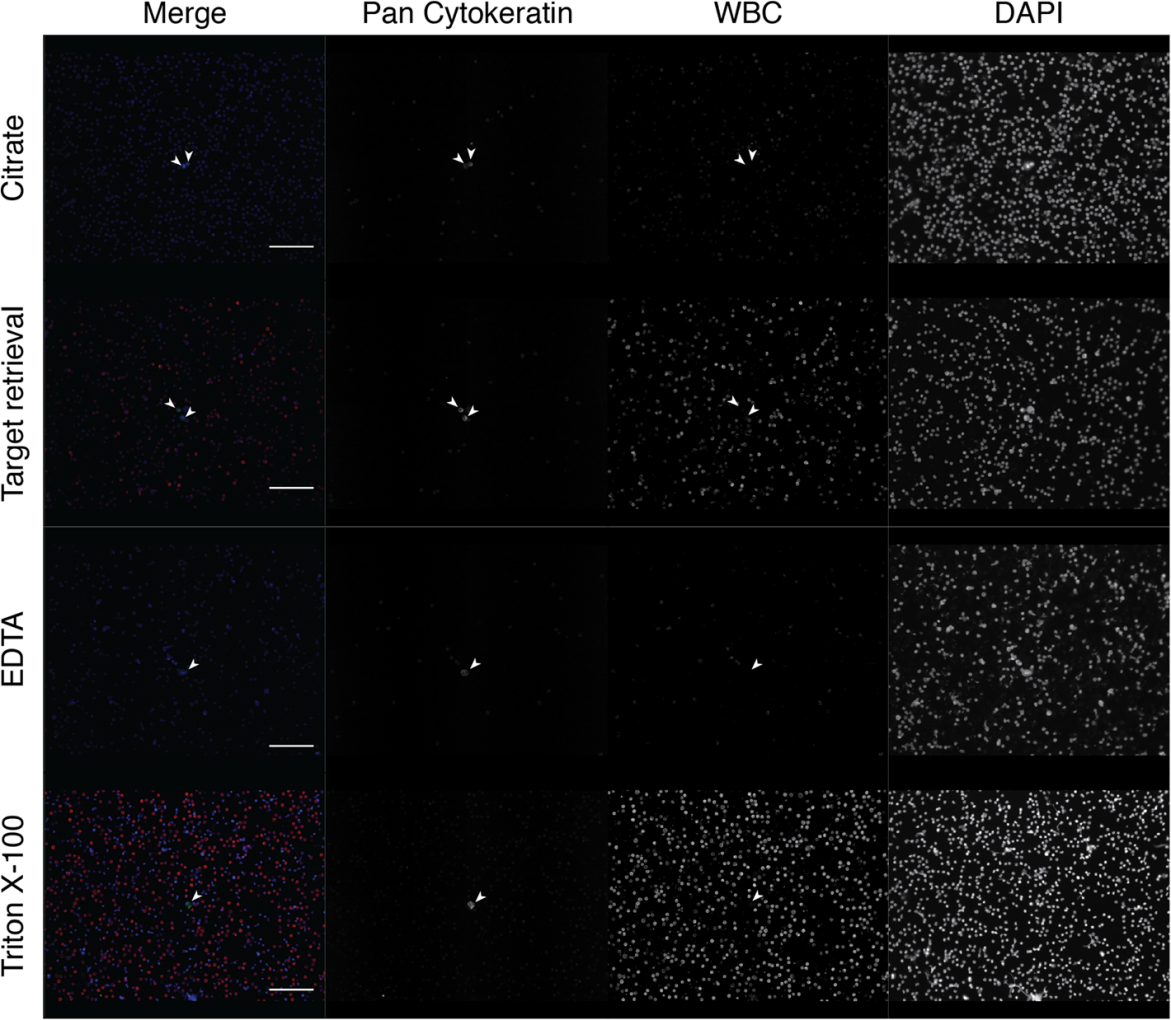
Comparison of antigen retrieval methods in cancer cell-spiked human bone marrow. Permeabilization using 0.5% Triton X-100 compared to heat-mediated antigen retrieval using Citrate-based Antigen Retrieval Solution, Target Retrieval Solution, and EDTA solution. White blood cell (WBC) staining represents a cocktail of mouse WBC antibodies (CD45, CD14, CD34, and CD66b) detected by one anti-mouse secondary antibody. Filled white arrowheads point to true positive staining. Scale bar = 50 μm

Most automated staining procedures perform heat-mediated antigen retrieval in a citrate-based buffer, while in manual staining protocols Triton X-100 is commonly used for cell permeabilization in IF protocols for cells on slides. We compared Triton X-100 to heat-mediated antigen retrieval with three different buffers to determine which produced the best signal-to-noise ratio. We found that permeabilization by Triton X-100 resulted in the best signal-to-noise ratio, as heat-mediated antigen retrieval with any of the buffers produced significantly greater background signal and had decreased true staining intensity (Fig. 2). Furthermore, antigen retrieval with EDTA buffer and target retrieval solution was harsh on the cells, resulting in cell loss and misshapen nuclei.

### Blocking Optimization

**Fig. 3:**
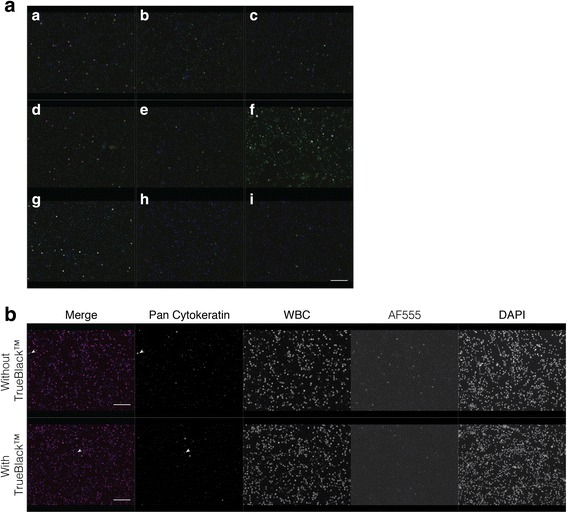
Optimization of blocking strategy to decrease background signal in cancer cell-spiked human bone marrow. **a** Comparison of different blocking reagents in primary antibody unstained samples. Goat anti-rabbit AlexaFluor 488 (AF488) and goat anti-mouse AF647 secondary antibodies were applied. Images display merged signal from each fluorescence channel. Blocking reagents used were a: 10% goat serum; b: 10% human serum; c: 5% BSA; d: 10% goat serum with 5% BSA; e: 10% human serum with 5% BSA; f: Protein Block Serum Free; g: SuperBlock™; h: Image-iT™ FX Signal Enhancer; i: BlockAid™. **b** Effect of TrueBlack™ on background signal due to autofluorescence. Samples were stained with pan cytokeratin and white blood cell markers. Autofluorescent signal reduction is most evident in the AF555 channel (where no primary or secondary antibody was applied) and in the AF488 channel (stained for pan cytokeratin). Filled white arrowheads point to true positive staining. Scale bar = 50 μm

The occurrence of background staining after secondary antibody addition indicated an insufficiency in blocking. Our original protocol used 5% goat serum for blocking, and while this has proven to be sufficient in the detection of CTCs and DTCs in mouse xenograft models, it has been reported that goat serum is ineffective for blocking human samples [22]. Thus, we compared other blocking reagents to improve upon this. In human BM samples stained only with secondary antibody (primary unstained samples), we determined that the best blocking strategies included Image-iT™ FX Signal Enhancer, 5% BSA, and BlockAid™ (Fig. 3a). However, when we used Image-iT™ Signal Enhancer for the blocking step and BlockAid™ as the antibody diluent (as suggested by the supplier), the background staining increased dramatically (data not shown). We concluded that the combination of an initial blocking step using Image-iT™ FX Signal Enhancer followed by a 5% BSA block step with 5% BSA as the primary antibody diluent was the most effective at limiting background signal. Reports have indicated that autofluorescence due to lipofuscin present in macrophages can also contribute to background signal [10–12], so we tested an additional blocking step using TrueBlack™, which quenches lipofuscin-related autofluorescence. Addition of a TrueBlack™ blocking step reduced autofluorescence particularly in the AF555 channel (Fig. 3b).

### Secondary Antibody Optimization

**Fig. 4:**
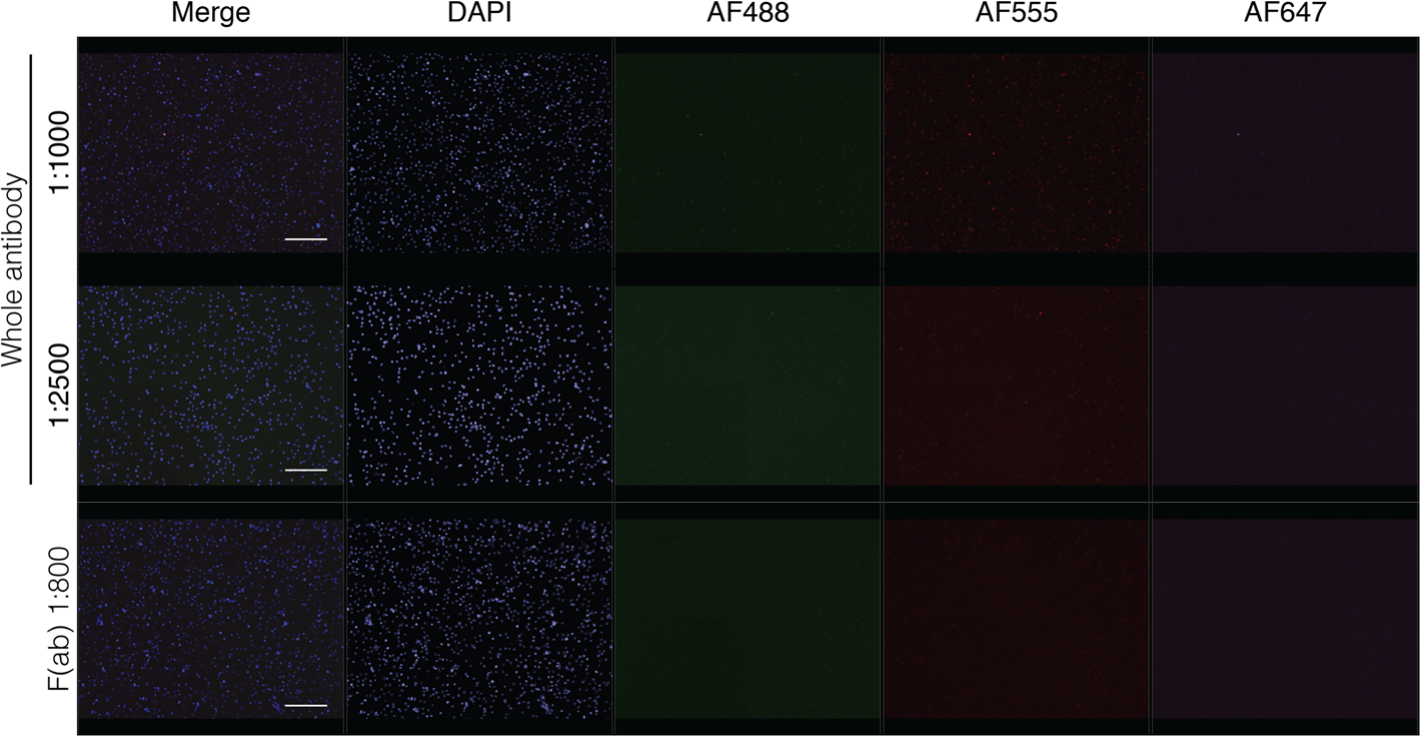
Optimization of secondary antibody application in human bone marrow. A) Comparison of different dilutions of whole secondary antibody and F(ab) fragment secondary antibody in primary unstained bone marrow. Goat anti-rabbit secondary antibodies for all three fluorophores were applied. Scale bar = 50 μm

Even though our optimized combination of blocking steps significantly reduced background, there was still a population of BM cells that bound secondary antibodies in the absence of a primary antibody. We hypothesized that this was due to Fc receptor binding, but addition of an Fc blocker did not completely eliminate this non-specific staining (data not shown). We were able to further reduce this background by diluting the secondary antibody; however, this did not completely eliminate the false signal (Fig. 4) and also decreased true positive signal (Additional file 2: Figure S1). We also reasoned that some non-specific staining could be due to secondary antibody aggregation facilitated by its trimeric structure. When we used pepsin-digested F(ab) fragment antibodies without the Fc portion, the background signal was further diminished (Fig. 4).

### Performance of the Optimized Protocol

**Fig. 5:**
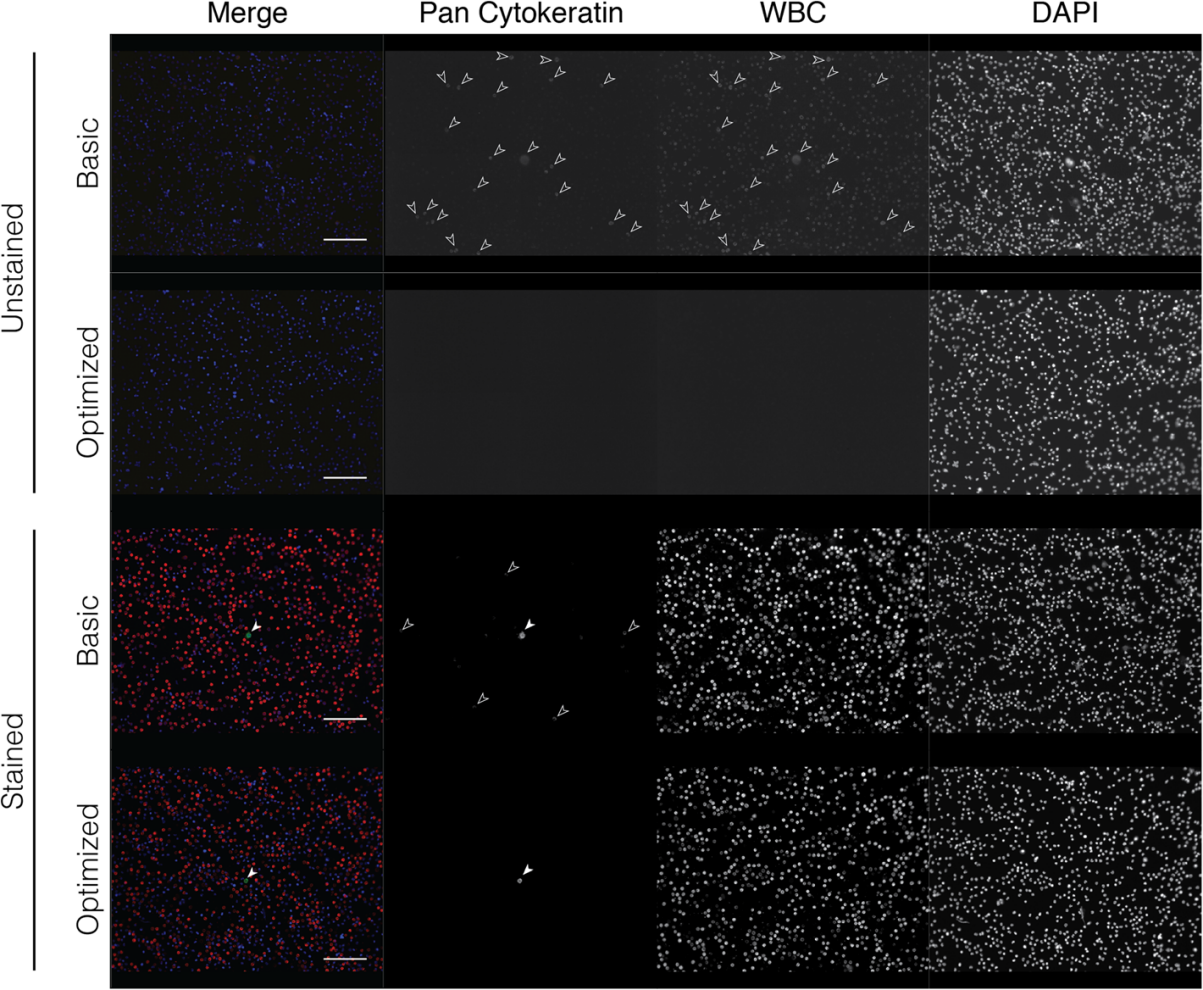
Comparison of the optimized protocol to the basic protocol. Cancer cell-spiked bone marrow was stained with (stained) and without (unstained) primary antibody using the basic or optimized protocol. Image exposure time was increased for primary unstained slides compared to stained slides, but was consistent between basic and optimized protocols for each. Filled white arrowheads point to true positive staining. Open solid arrowheads indicate false staining due to non-specific binding of secondary antibody. Scale bar = 50 μm

**Fig. 6:**
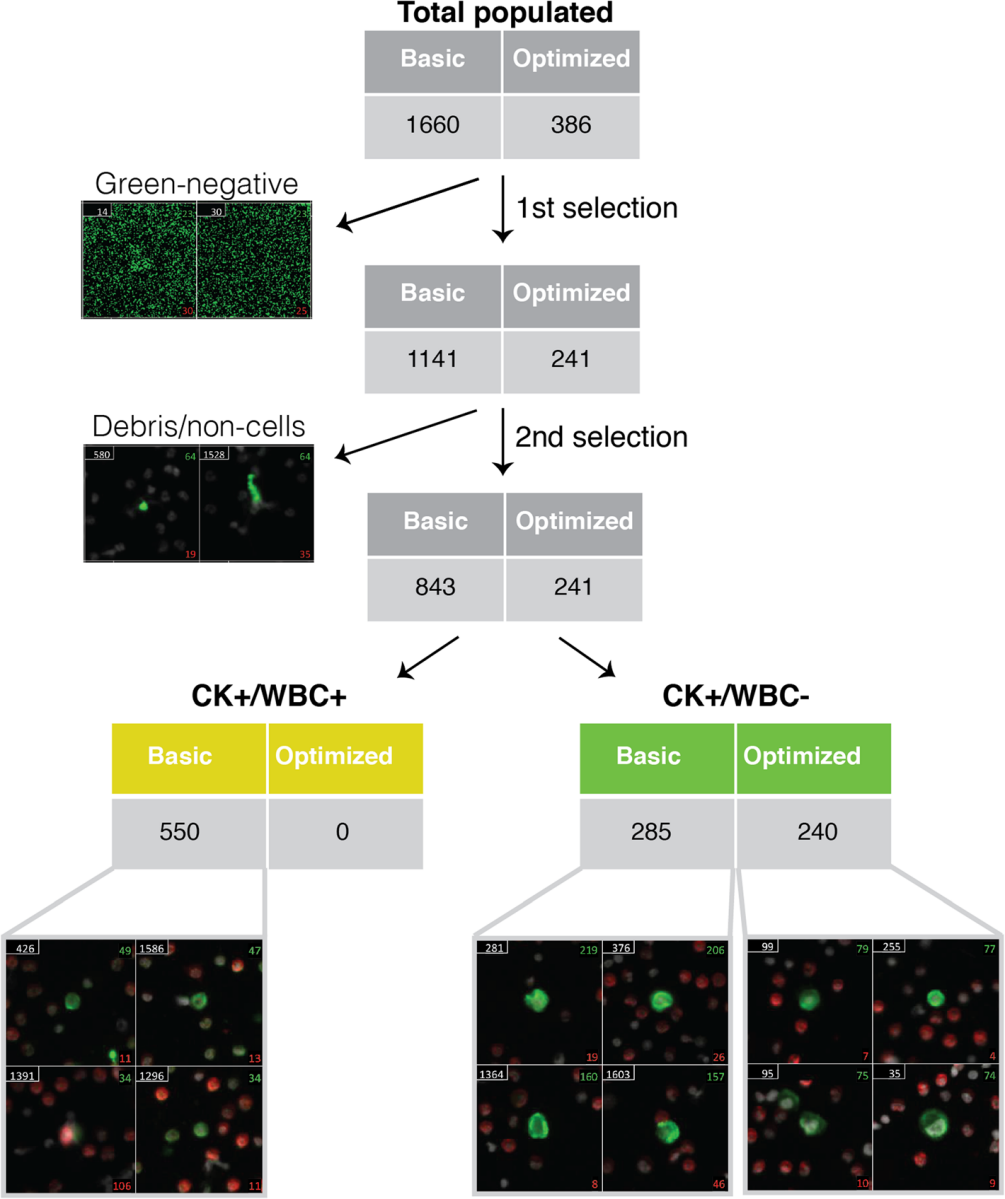
The optimized protocol enhances detection capability of automated software as compared to the basic protocol. Comparison of the number of pan cytokeratin+ and white blood cell+ (CK + WBC+) cells detected within a defined area with automated scanning software using the basic and optimized protocols for primary stained, cancer cell-spiked slides from 5A. After the software generated a gallery of green-positive cells (CK+ candidates; total populated), the gallery was manually scanned to eliminate green-negative events (background signal only due to high exposure time; first selection) and then a second selection was applied to eliminate debris and non-cellular events. This left a gallery with candidate cancer cells (CK + WBC-) and double positive WBCs with false CK signal (CK + WBC+)

**Fig. 7:**
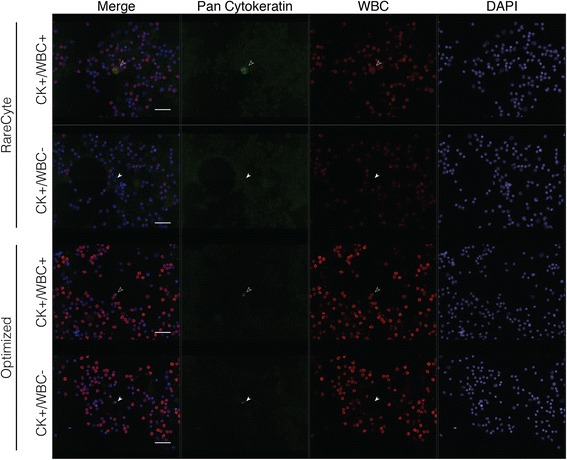
The optimized protocol enhances detection of patient DTCs compared to alternately stained and processed slides. Selected images representing candidate disseminated tumor cells (CK + WBC-) and CK + WBC+ cells from Patient 1 in Table 1 stained with the RareCyte or optimized protocol. Image exposure time and brightness and contrast settings had to be altered between the slides stained with the optimized and the RareCyte protocol due to a decrease in overall signal with the RareCyte protocol. Scale bar = 40 μm

After optimizing every step of the staining protocol, we directly compared it with the basic protocol in cancer cell-spiked and unspiked human BM. We also included primary antibody unstained samples to observe background staining. We found that background signal was significantly reduced using the optimized protocol, and that the overall signal-to-noise ratio was greater (Fig. 5). It was also easier to detect CK + WBC- cells by eye when manually scanning the slide. Since manual imaging can be easily manipulated to produce optimal signal, and is not feasible for analyzing large batches of slides to detect rare cells, we then used the Metafer scanning software to scan each slide and populate a gallery of CK+ candidate cancer cells (green) (Fig. 6). For each slide we used the same scan settings, in which we are able to control the maximum and minimum exposure time for each field, and the signal intensity cutoff for populating the gallery. Exposure time never exceeded 120 ms, and we used lenient cutoff criteria so that low CK-expressing cells would still be detected. Once the gallery was generated we went through three rounds of selection criteria to get rid of extraneous events that the software picked up (Fig. 6). We first eliminated anything that did not visually show up as green. The number of events that was initially detected by the software was almost four times higher when the basic protocol was used, and was still higher after the first screening, indicating that the automated software was picking up background signal. We then eliminated anything that was clearly not a cell or was not actually green positive after manual observation of the cell. This left us with a gallery of cells that looked to have true positive staining for CK. However, when we assessed the WBC channel (AF555, red) there were cells that were double positive for red and green signal. This was only observed in the slide stained with the basic protocol, and there were more CK + WBC+ cells (putative WBCs) than CK + WBC- cancer cells (putative cancer cells). On the other hand, in the gallery from the slide that was stained with the optimized protocol, we were easily able to eliminate cells in the first screening step that were not green positive, resulting in only true CK + WBC- cancer cells and no double positives or even questionable staining. We then wanted to determine if our optimized protocol had advantages over other established rare cell detection protocols and was compatible with other BM processing techniques. We stained three BM aspirates collected near the site of a metastatic lesion from prostate cancer patients. These samples were collected and processed using the RareCyte AccuCyte® system [21]. One of each sample was stained using a manual version of the automated protocol used by RareCyte, which included vastly different reagents and steps compared to ours, or using our optimized protocol. Currently the RareCyte protocol is optimized for use only in blood to detect CTCs, and is not recommended for use in BM. Each slide was scanned using the Metafer detection software to compare the detection of CK + WBC- candidate DTCs between protocols for each patient (Table 1). The higher signal-to-noise ratio observed using the optimized protocol was evident by the decreased number of initial events populated by the gallery and a greater number of candidate DTCs (CK + WBC-) for every patient. Overall it was easier to detect candidate DTCs by eye with the optimized protocol, but due to low CK expression in the patient DTCs requiring high exposure times there remained cells that appeared CK + WBC+ for each protocol (Fig. 7). Our optimized protocol was more sensitive (39.3 average DTCs compared to 2.5 average DTCs) (Table 1) given that each slide for a particular metastatic patient should theoretically have the same number of DTCs.

**Table 1:** Cell Counts After Scanning Metastatic Patient BM Stained with the Optimized Protocol or RareCyte Protocol

Patient	Protocol	Total populated	First selection	Second selection	CK + WBC+	CK + WBC-
1	RareCyte	15,558	67	29	24	5
	Optimized	3775	77	71	51	20
2	RareCyte	> 200,000	N/A	N/A	N/A	N/A
	Optimized	7318	109	76	20	56
3	RareCyte	9849	84	40	40	0
	Optimized	2774	181	91	49	42

## Discussion

The ability to predict cancer recurrence before the development of overt metastatic lesions could prevent many deaths due to metastasis. Presently there are no reliable methods to detect recurrent disease before it becomes incurable, but there have been promising developments in liquid biopsy-based rare cell assays. Detection of rare cells by IF staining of BM smears not only allows for quantification, but allows for their biological characterization, which will be crucial in guiding treatment strategies. Unfortunately, there is no widely accepted staining protocol that has the necessary specificity and sensitivity criteria to accurately detect ~ 1 cancer cell out of ten million BM cells. It is imperative that procedures intended for clinical use like this be standardized and rigorously optimized, as they are meant to inform important diagnostic and prognostic decisions. The closest “gold standard” is an FDA-approved IF-based detection platform called CELLSEARCH [6]. However, it is only approved for CTC detection in blood and relies solely on the epithelial markers EpCAM and cytokeratin. While the detection of CTCs using other markers and platforms have also been somewhat successful, no method for BM DTC detection has been successful [5]. Since it is more informative to understand the biology of a “successful DTC” in the BM compared to the many hundreds of CTCs that enter the circulation but do not survive, it is essential that a standard procedure for IF staining of the BM be developed [23]. The lack of platforms for DTC detection is due to the increased immune component, increased autofluorescence, and difficulty of staining samples in the BM compared to in the blood. The consequences of this are two-fold: 1) more immune cells produce more false positive staining, and 2) markers used to detect CTCs may no longer be specific for DTCs because they might also be expressed by a particular population of BM cells. In order to accurately assess specific detection markers for rare cells, it is critical that any false staining attributed to the autofluorescent nature and large immune component of the BM be eliminated.

In our experience with staining BM for CK to detect cancer cells, we have found that primary antibody unstained cancer-free samples contain cells that have positive signal and are indistinguishable from real signal in cancer cell line spiked samples by automated detection software. It is possible that some BM cells can express CK; however, the positive control cancer cells used here are distinguishable by their high frequency and staining pattern. The cells found in cancer-negative controls display spatially overlapping signal in each channel with no distinct pattern, whereas true cytoplasmic filamentous CK staining should only be detectable in one pre-determined channel. In this study, we were able to identify and eliminate the sources contributing to the ambiguous background signal: autofluorescence and non-specific binding of fluorescently-labeled secondary antibody. Other studies have observed these background signals in BM as well, citing macrophages and neutrophils as major culprits [9, 24]. Both sources of background were visible in every channel, but autofluorescence was the strongest in the AF555 channel. We were able to decrease this BM tissue autofluorescence by reducing fixation time from 30 to 10 min, including a TrueBlack™ blocking step, and using Triton X-100 permeabilization over heat-mediated antigen retrieval methods. To reduce non-specific binding of secondary antibodies (the main source of strong background signal), we combined blocking with 5% BSA and Fc receptor block with the Image-iT™ Signal Enhancer. To amplify true signal we used indirect labeling with primary and secondary antibodies rather than fluorophore-conjugated primary antibodies. We found that the use of F(ab) fragment secondary antibodies was helpful in reducing background signal for CK, but that the use of whole secondary antibodies boosted the signal of WBCs (Additional file 2: Figure S1). These steps collectively increased true signal and reduced background signal, allowing for shorter exposure time and a higher signal-to-noise ratio. Other conditions we found to enhance signal-to-noise ratio but that we did not test directly included washing and antibody incubation temperature and time. We extended wash times and used PBST instead of PBS, and performed all antibody incubations at room temperature instead of at 37 °C, which was previously recommended [18]. By changing these parameters, we were able to eliminate all significant background contributing to high false CK signal on immune cells.

Due to the rarity of DTCs, the use of automated scanning microscopy is required for their detection and enumeration. We compared our novel optimized IF protocol to our previous basic IF protocol, as well as to a CTC detection protocol from RareCyte. When we stained cancer cell-spiked BM with our optimized protocol, we were able to detect the same number of CK + WBC- putative cancer cells as the basic protocol, but with a significant reduction in manual screening time (ten minutes compared to three hours). This was due to an enormous reduction in extraneous events, caused by high background. When we stained advanced cancer patient BM with our optimized protocol compared to the RareCyte protocol (optimized for blood, not BM), we detected more CK + WBC- putative DTCs. We also observed an impressive reduction in background signal, which allowed for significantly less manual screening time. In these patient samples we observed more extraneous events and CK + WBC+ cells than in the cell-line spiked BM (Table 1); this is likely due to lower CK expression in the actual patient DTCs relative to the cancer cell line, so a much higher exposure time was required. In some cancers, however, CK expression is considerably higher, so the signal-to-noise ratio is much greater (Additional file 3: Figure S2). CK heterogeneity was also observed between cancer cells in the cancer cell line and in patient samples. The purpose of our study was to eliminate background signal contributing to inconsistencies and uncertainty when testing cancer-specific markers. To this end, our optimized protocol 1) increased signal-to-noise, 2) decreased extraneous events picked up by automated detection software, 3) can be applied to variously prepared samples, and 4) detected patient DTCs. Ongoing work is being done to adapt this optimized protocol for use with automatic staining devices for high volume processing, a requirement for rare cell detection.

## Conclusions

In this study, we have optimized the staining conditions for BM smears in order to control and minimize signal due to factors other than the expression of the candidate cancer cell marker. Our optimization increased the signal-to-noise ratio and eliminated sources of false staining. This assay can now be used to investigate the sensitivity and specificity of individual candidate disease-specific markers (e.g. prostate specific antigen for prostate cancer), as any observed signal can be confidently qualified as a true positive signal. Finally, we are working to automate this assay for use with high volume clinical samples in order to detect and characterize CTCs and DTCs to predict cancer recurrence.

## Additional files

Additional file 1: **Tables S1-S3.** (DOCX 15 kb)

Additional file 2: **Figure S1.** (PNG 1806 kb)

Additional file 3: **Figure S2.** (PNG 583 kb)

## Abbreviations

BM: Bone marrow
CTC: Circulating tumor cell
DTC: Disseminated tumor cell
Fc: Fragment crystallizable
IF: Immunofluorescence
WBC: White blood cell
