# Supplementary material for: Optimization of Immunofluorescent Detection of Bone Marrow Disseminated Tumor Cells
Source: Biol Proced Online. 2018 Jul 1;20:13. doi: 10.1186/s12575-018-0078-5 (PMC6026516; doi:10.1186/s12575-018-0078-5)
Supplement: Supplementary file 1 — Tables S1-S3. (DOCX 15 kb) [file 12575_2018_78_MOESM1_ESM.docx]

| **Table S1: Reagents used for IF** | | |
| --- | --- | --- |
| **Reagent** | **Company** | **Catalog** |
| Triton X-100 | Fisher Scientific | BP151-500 |
| 10X PBS pH 7.4 | Quality Biological | 119-069-131CS |
| Tween-20 | Sigma | P9416 |
| 10% Normal Goat serum | ThermoFisher | 50062Z |
| Human Serum from male AB plasma | Sigma | H4522 |
| Serum-Free Protein Block | Dako | X0909 |
| SuperBlock (TBS) | ThermoFisher | 37535 |
| BlockAid™ Blocking solution | ThermoFisher | B10710 |
| Image-iT™ FX Signal Enhancer | ThermoFisher | I36933 |
| BSA | Fisher Scientific | BP1600-1 |
| TrueBlack™ Lipofuscin Autofluorescence Quencher | Chemometech | 23007 |
| Human TruStain FcX™ (Fc Receptor Blocking Solution) | BioLegend | 422301 |
| ProLong™ Gold Antifade Mountant with DAPI | ThermoFisher | P36931 |
| ProLong™ Gold Antifade Mountant | ThermoFisher | P36930 |
| Anti-Pan-Cytokeratin | Abcam | Ab9377 |
| Anti-CD45 | Abcam | Ab8216 |
| Anti-CD14 | BioLegend | 301802 |
| Anti-CD34 | BioLegend | 343501 |
| Anti-CD66b | BioLegend | 305102 |
| Goat anti-Rabbit AffiniPure F(ab) IgG (H+L) Fragment AF488 | Jackson ImmunoResearch | 111-547-003 |
| Goat anti-Rabbit IgG (H+L) AF488 | ThermoFisher | A11034 |
| Goat anti-Mouse IgG (H+L) AF555 | ThermoFisher | A32727 |
| Goat anti-Mouse IgG (H+L) AF647 | ThermoFisher | A32728 |
| Moisture chamber | Evergreen | 240-9020-Z10 |
| Vertical glass staining dish | Fisher Scientific | 08-815 |

| **Table S2: Clinical information for BM samples collected for processing onto adhesion slides** | | | |
| --- | --- | --- | --- |
| **JHBUI** | **Clinical TNM stage** | **Biopsy Gleason** | **PSA** |
| 4294 | T1c Nx Mx | 3+3 | 5.5 |
| 4322 | T1c Nx Mx | 3+3 | 6.02 |
| 4392 | T1c Nx Mx | 3+4 | 4.1 |
| 4477 | T1c N0 M0 | 3+4 | 28.0 |
| 4537 | T1c N0 M0 | 3+3 | 5.9 |
| 4969 | T1c N0 M0 | 3+3 | 4.8 |

**Table S3: Clinical information for metastatic bone marrow samples collected for processing onto plus slides.**

| **Table S3: Clinical information for metastatic BM samples collected for processing onto plus slides** | | | | | |
| --- | --- | --- | --- | --- | --- |
| **Patient** | **Collection site** | **Metastatic site** | **Clinical TNM stage** | **Biopsy Gleason** | **PSA** |
| 1 | L Iliac crest | R proximal femur, L inferior scapula, posterior calvarium | cN1 cM1 | 4+3 | 154 |
| 2 | R Iliac crest | L pelvic LN | cT1c cN1 cM1 | 5+4 | 26 |
| 3 | T11 (thoracic spine) | C6, T5, T7, L1, L2, and L3 of the spine; L 7th rib | cT2b cN1 cM0 | 3+3 | 51 |
