# Supplementary figures and images for: Optimization of Immunofluorescent Detection of Bone Marrow Disseminated Tumor Cells
Source: Biol Proced Online. 2018 Jul 1;20:13. doi: 10.1186/s12575-018-0078-5 (PMC6026516; doi:10.1186/s12575-018-0078-5)

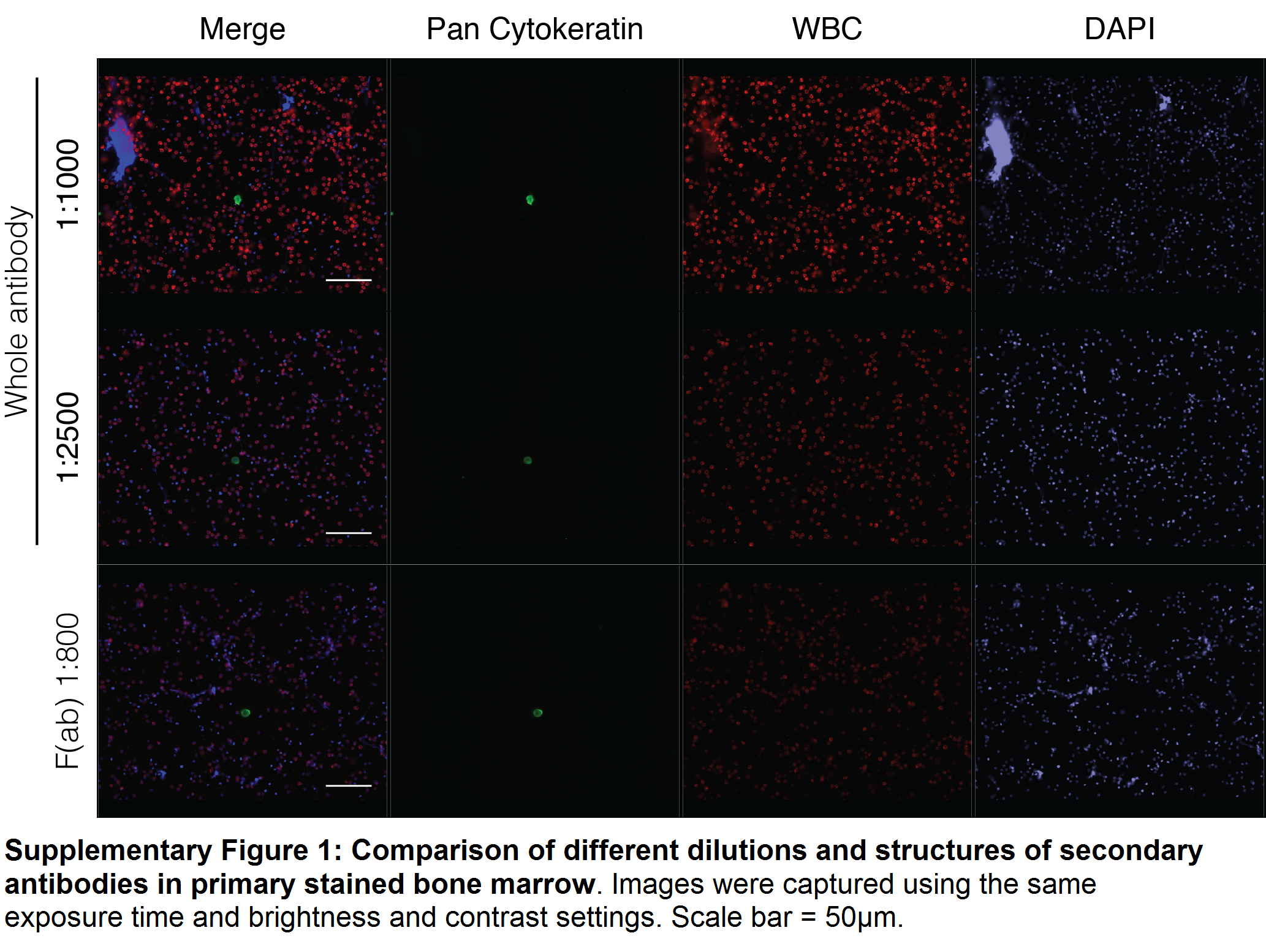

Supplement: Supplementary file 2 — Figure S1. (PNG 1806 kb) [file 12575_2018_78_MOESM2_ESM.png]

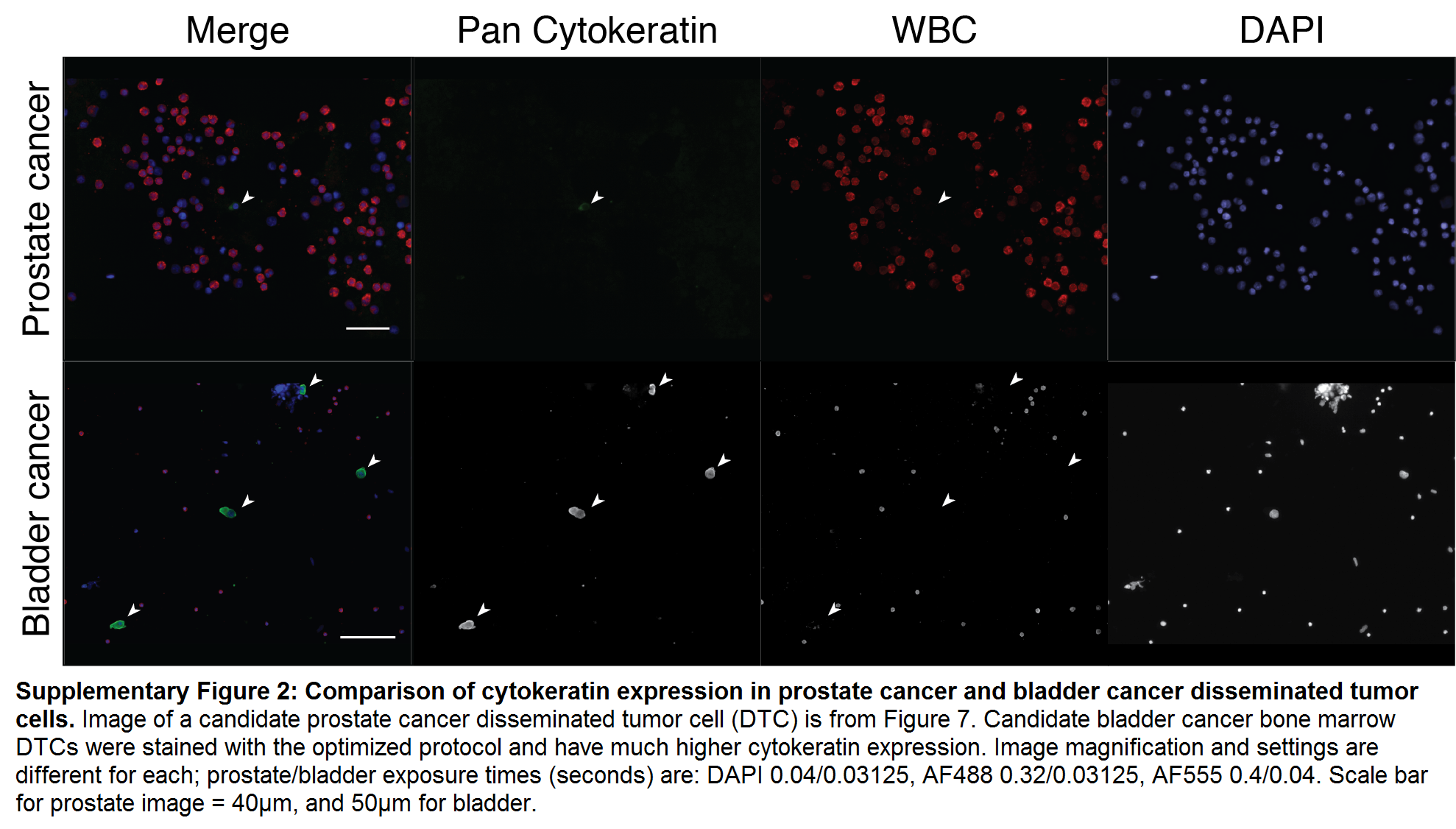

Supplement: Supplementary file 3 — Figure S2. (PNG 583 kb) [file 12575_2018_78_MOESM3_ESM.png]
